# Making Medical History Relevant to Medical Students: The First Fifty Years of the Calgary History of Medicine Program and History of Medicine Days Conferences

**DOI:** 10.1093/jhmas/jrac044

**Published:** 2023-01-04

**Authors:** Frank W Stahnisch

**Affiliations:** University of Calgary, Alberta, Canada

**Keywords:** Canada, clinic-based teaching of history, History of Medicine Days student conferences, history of medicine programs, medical education, University of Calgary

## Abstract

Medical historians and educators have long lamented that the integration of the study of the history of medicine into the educational curricula of medical schools and clinic-based teaching has been protractedly troubled. Employing the development of the history of medicine program at the University of Calgary as a case study, this article emphasizes the importance of integrating medical history with teaching schedules to further students’ insights into changing health care settings, the social contingency of disease concepts, and socio-economic dependences of medical decision-making. History of medicine programs can furnish plentiful opportunities for research training through summer projects, insight courses, and field *practica.* This article explores the first fifty years of the History of Medicine and Health Care Program in Calgary and considers the impact of interdisciplinary cooperation as well as the role of interprofessional undergraduate and clinical medical education. Through this exploration, I argue that medical history should be a central part of study curricula, that a historical understanding can provide a robust background for physicians in a fast-changing world in the clinic, and that through their disciplinary expertise, medical historians play a fruitful role in scholarly and teaching exchanges with medical students and clinicians in the modern medical humanities.

Karl Sudhoff’s (1853–1938) institute for the history of medicine, which opened in 1906 at the University of Leipzig in Germany, has long been considered a gold standard for history of medicine programs affiliated with medical faculties.^1^ The Leipzig program considered the history of medicine to be firmly within the realm of medical students and clinical trainees themselves. Medical history has since become an inspirational academic field in medical schools across Europe, North America, and around the world.^2^ It enthralled medical students, fostering work by graduate students, as well as initiating collaborations among clinical faculty, biomedical researchers, and public health workers.^3^ One such program is the thriving history of medicine community at the University of Calgary with its History of Medicine and Health Care Program (HOMHCP) in Canada, established with the help of the Jason A. Hannah Foundation – now known as Associated Medical Services (AMS) – and the Alberta Medical Foundation (AMF).^4^

A statue of Hippocrates stands in the Health Sciences Centre (HSC) of the University of Calgary’s (UofC’s) Cumming School of Medicine (CSM). It is a legacy of Peter J. E.Cruse’s (1927–2006) history of medicine courses and was a gift from the sizeable Greek-Canadian population to the UofC’s new medical school.^5^ This marble statue arrived from Greece in 1973 (see figure 1) and was installed at a prominent place in the foyer of the HSC, where it remains a popular meeting location, a site of educational commemoration, as well as a place that sees rites of passage in medical students and residents happening to this day.

**Figure 1.**
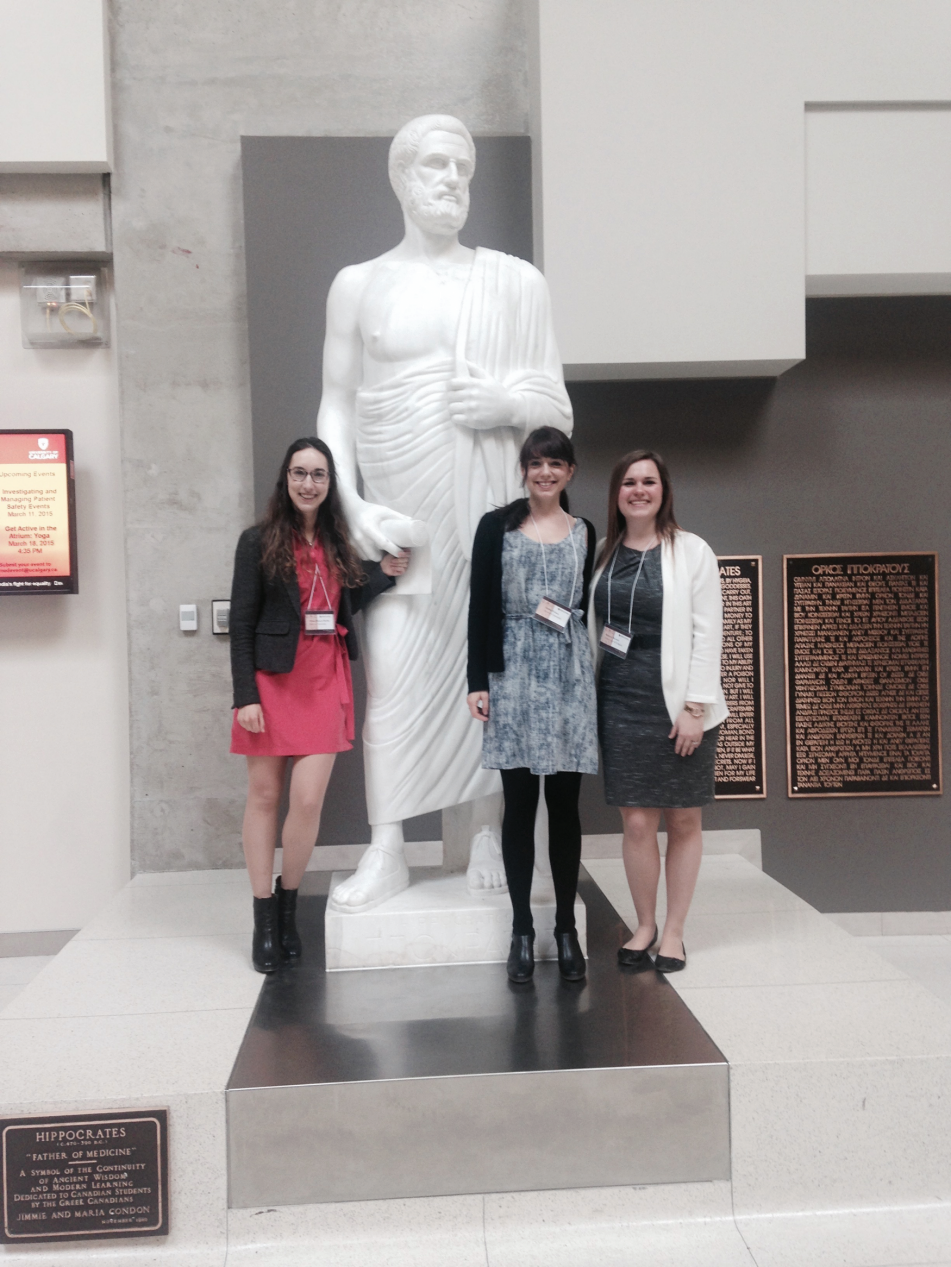
Queens Group of Medical Students at the Hippocrates Statue, HMDs, 2015. Courtesy of Dr. Jacalyn Duffin, Queen’s University, Kingston.

## A NEW MEDICAL SCHOOL IN CALGARY

Founding faculty member Dr. Cruse pushed for the inclusion of medical history in the schedule of Calgary’s new medical school (1970–1971), from which workshops emerged for residents in surgery and staff surgeons.^6^ It began with 7:00 AM discussions on clinical topics, such as physicianship, the development of operational techniques, and infection control in historical contexts. Cruse’s charismatic advocacy for the subject matter rendered the sessions into an appealing event.^7^ The discussions evolved into a popular elective, that realized Cruse’s intention to fuse the art and science of medicine by teaching about past exemplars, such as the French surgeons of the eighteenth century, advances in surgical technique by late nineteenth-century German surgeons, or the creators of specialized surgical fields in twentieth-century America.^8^ The program was gradually extended to all medical students by the end of the 1970s and expanded nationally in 1991 with the two-day History of Medicine Day Conferences (HMDs) (see table 4).^9^ In 1996, a third day was added, when the students travelled to the scenic Rocky Mountains after the conference deliberations.^10^

Calgary’s founding physician-historian Cruse was born in Stellenbosch, South Africa. His father, Henri P. Cruse (1894–1950) was a university professor specializing in the history of pedagogy at Stellenbosch University, while his mother Aletta (1897–1965?) worked as a schoolteacher.^11^ After his studies at the University of Cape Town, his M.B. Ch.B. graduation in 1951, and completion of the FRCS(E) in Edinburgh, UK, the family moved to Canada.^12^ Earle P. Scarlett (1896–1982), who had received his training at the Universities of Manitoba and Toronto and completed his residency with William Osler (1849–1919) in Montreal, became Cruse’s mentor in Calgary.^13^ When the new Foothills Medical Centre (FMC) opened, Cruse received a surgery position there and eventually rose through the ranks of the newly founded medical school, becoming a full professor in 1975.^14^ Even with his time-intensive work in medical history and infection control research, his main career focus was that of a prolific surgeon as evidenced through his role as head of the surgery department from 1981 to 1988.^15^ Additionally, he continued his academic interchanges with Scarlett, who was by that time a board member of the hospital.^16^ Cruse shared Scarlett’s expectation that participating students in the history of medicine course would write a research paper on a subject related to their academic work and join excursions related to the course in Calgary, from which they would get insights into the social and historical contexts of medical practice. They would write scholarly papers under supervision of faculty preceptors and gain experience in speaking in front of their classmates, large crowds at the HMDs conferences, and at gatherings of the annual general meetings of the Alberta Medical Association (AMA).^17^ This process enabled journal publication of the projects, continuing the tradition of Scarlett’s *The Calgary Associate Clinic’s Historical Bulletin*. The Bulletin, as it was previously called, was published between 1936 and 1958, focusing greatly on the history of medicine in Alberta, but also on the history of medicine in general, in 22 volumes comprising 88 issues.^18^ Scarlett’s Bulletin also influenced the early founders of the flagship *Canadian Bulletin of Medical History* (*CBMH)* when it was created as the newsletter of the Canadian Society for the History of Medicine in 1979. Published twice a year for the Canadian Society for the History of Medicine, originally by Wilfrid Laurier University Press and now through the University of Toronto Press, the *CBMH* has prominently featured several research articles from faculty and trainees participating in Calgary’s HOMHCP.^19^

**Figure 2.**
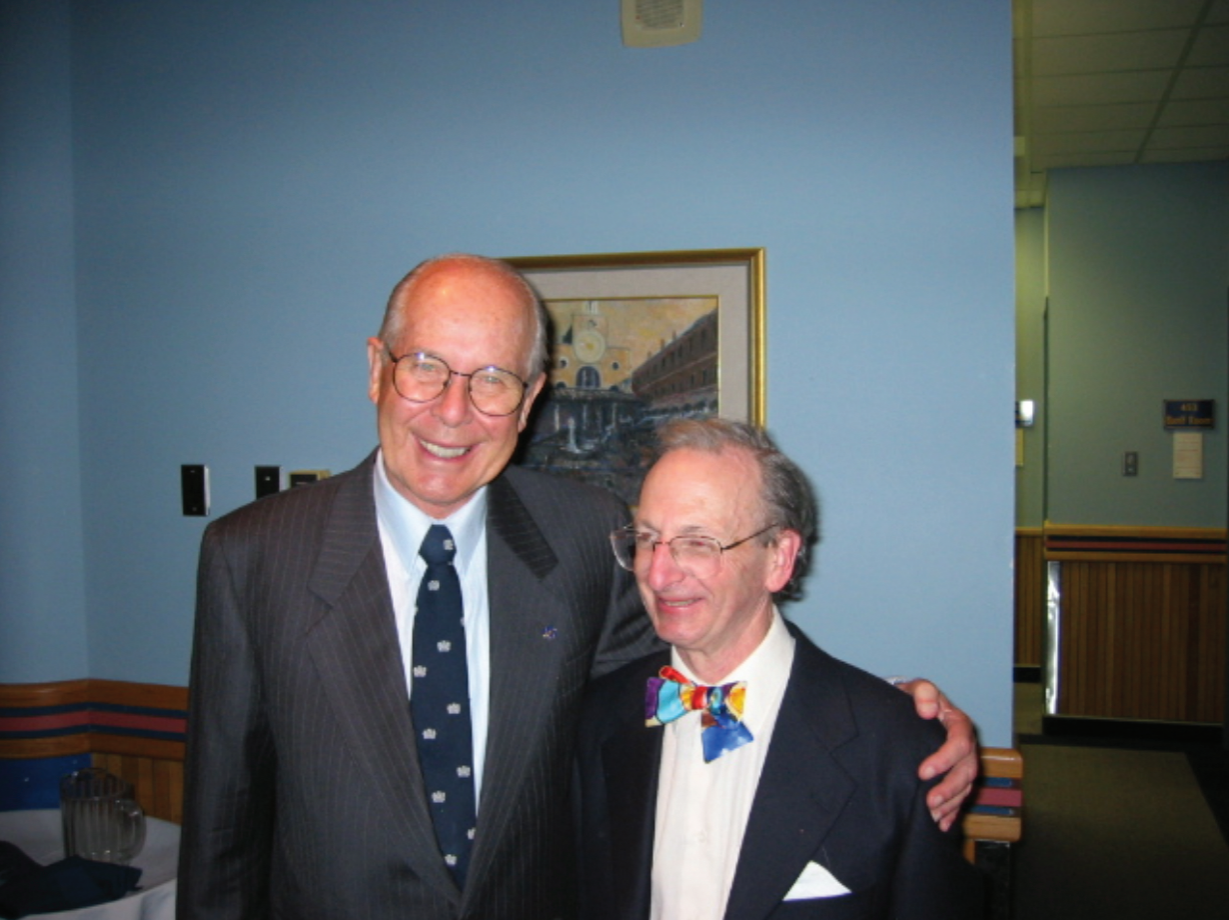
The two founders of the Calgary history of medicine program: surgeon Dr. Peter J. E. Cruse (on the left) and internist Dr. William A. Whitelaw (on the right) at a monthly meeting of CHOMS (1990s). Photograph courtesy of the late Dr. Peter Warren (1940–2011), University of Manitoba, Winnipeg.

Cruse himself successfully rendered history of medicine even more popular by arranging to take his medical students to the meetings of the Royal College of Physicians and Surgeons of Canada (RCPSC), where they gave short history of medicine presentations to the assembly.^20^ In acknowledgment of his achievements, AMS recognized him as the Canadian Medical Historian of the Year in 1989.^21^ That summer he also approached the AMF to sponsor a HMD at the UofC, which would showcase presentations of the best papers from the history of medicine course. This accomplished, Cruse’s students were soon joined by the medical history students of Laurie Clein (d. 1996) at the nearby University of Saskatchewan, which has continued to this day with strong regional contributions from the Universities of Manitoba, Regina, Lethbridge, Alberta, British Columbia, and Mount Royal University. Financial support from AMS in Toronto and AMF in Edmonton eventually permitted the creation of the AMF/Hannah Professorship in the History of Medicine and Health Care in 1995.^22^ With Cruse’s decreasing health situation, the helm was passed on to McGill-trained internist and history *aficionado* Bill Whitelaw, who headed the conference until 2007 (see figure 2).^23^

Presentation topics at the HMDs have covered areas such as the classics (including the Hippocratic Oath and changes in the social standing of the physician), history of public health (e.g., different measures against epidemics, training, and human resources), nursing (including the interdisciplinary nature of modern medicine and the physician-nurse relationship), veterinary medicine (e.g., the status and ethics of animal experimentation and collaborations in laboratory science), as well as neuroscience (including the clinical and scientific implications of the mind-body dualism) (see table 2). Prizes, which have also emphasized the clinical relevance of the presentations, have been awarded in different categories that fostered medical students’ interest in the history of medicine and enticed them further to produce high-quality presentations and posters. These awards included history of surgery (Dr. Peter Cruse Award), internal medicine (Dr. Bill Whitelaw Award), women in medicine and public health (Dr. Clara Christie Award), best poster award, best audiovisual presentation, and the best overall conference presentation (see table 3).^24^ Medical students and residents, with their clinical experiences from the hospital wards, have been specifically targeted and requested to present original historical research with new methodological perspectives. The conference sections have been moderated by historians of medicine and psychology, classicists, as well as historically interested clinical faculty and surgeons from the FMC, the Peter Lougheed Hospital, the Rockyview General Hospital, and the Alberta Children’s Hospital, who have all been eager to engage with the medical students and to integrate the insights gained from the history of medicine sessions in their own clinical work.^25^ Internationally renowned speakers, with a balanced proportion between clinician-historians and professional medical historians, are invited to give keynote lectures and engage with trainee presenters at the conferences.^26^ Beginning in 2009, a specialized, reviewed series with *Cambridge Scholars Publishing* in Britain has emerged to document the conference proceedings and include the best peer-reviewed research papers given at the conferences. These publications combine the submitted and accepted papers regarding clinical medical history topics, as well as those topics in health care history by other interested undergraduate and graduate students from across Canada. It is also a welcome opportunity for medical residents to write and publish some of the first papers in their clinical careers. Of all the submitted conference papers, up to fifteen of the best papers are considered for publication in the conference proceedings, often making substantive contributions to our medical understanding and the existing historiography, including for example original work on the early ambulatory and mobile clinical stations in Atlantic Canada, the introduction of super glue into modern surgery, and assessments of the economy of material culture of menstruation products in public health and gynaecology to mention here only a few.^27^

**Table 1:**
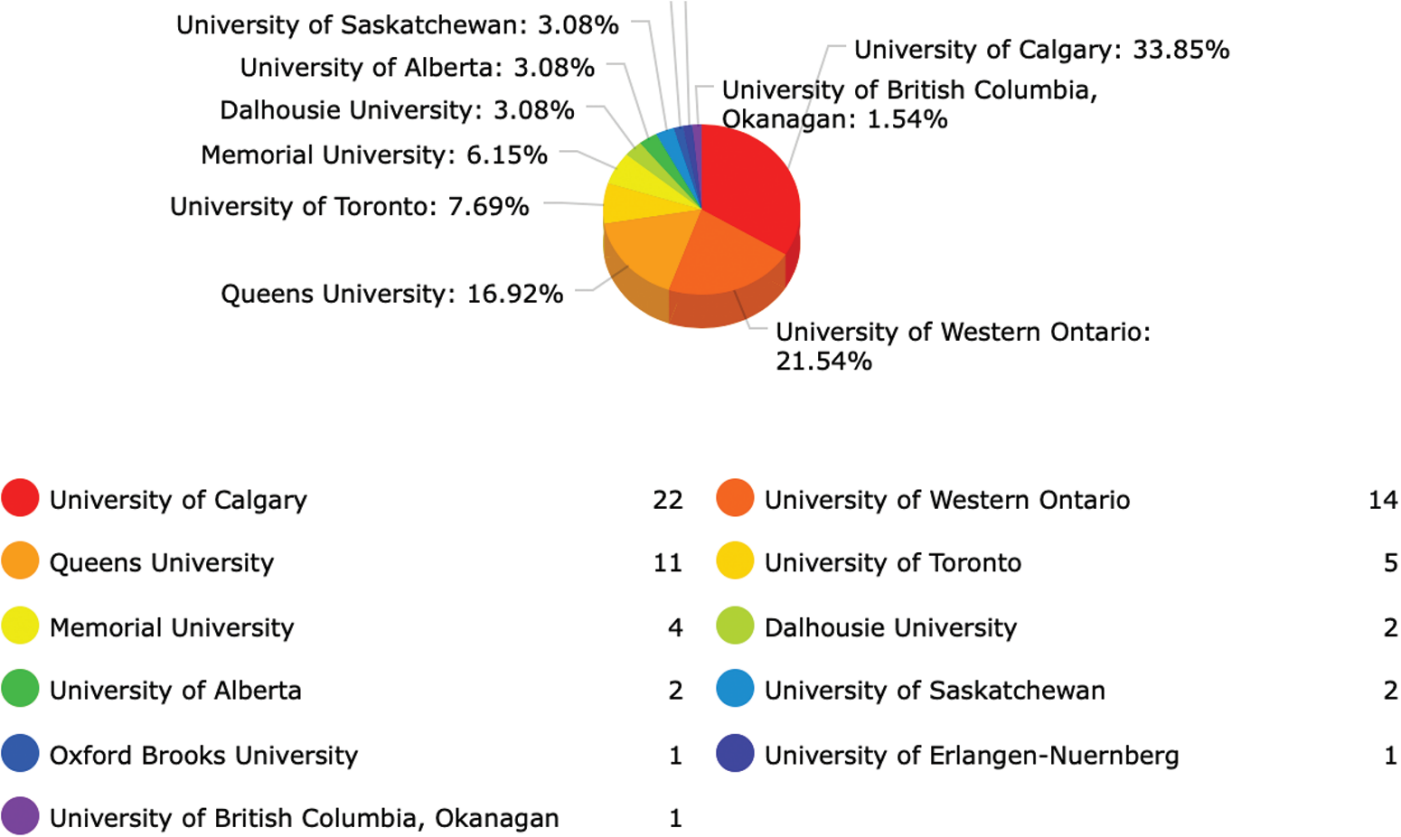
Medical Schools and Universitites of Origin of Active HMDs Presenters (above), including Numbers of Years Participated (below).

**Table 2.**
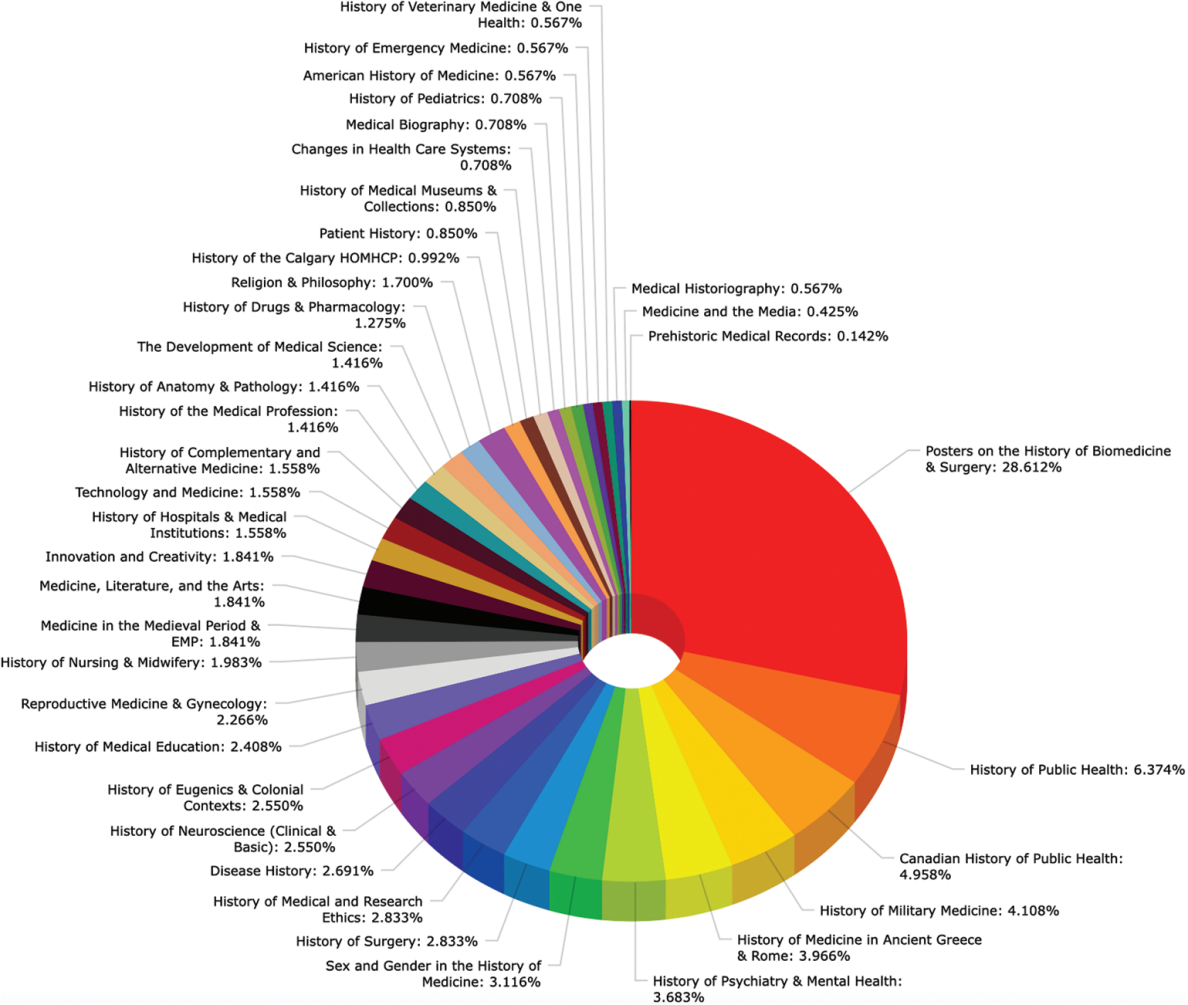
Percentage of Topics Presented at the HMDs.

**Table 3.**
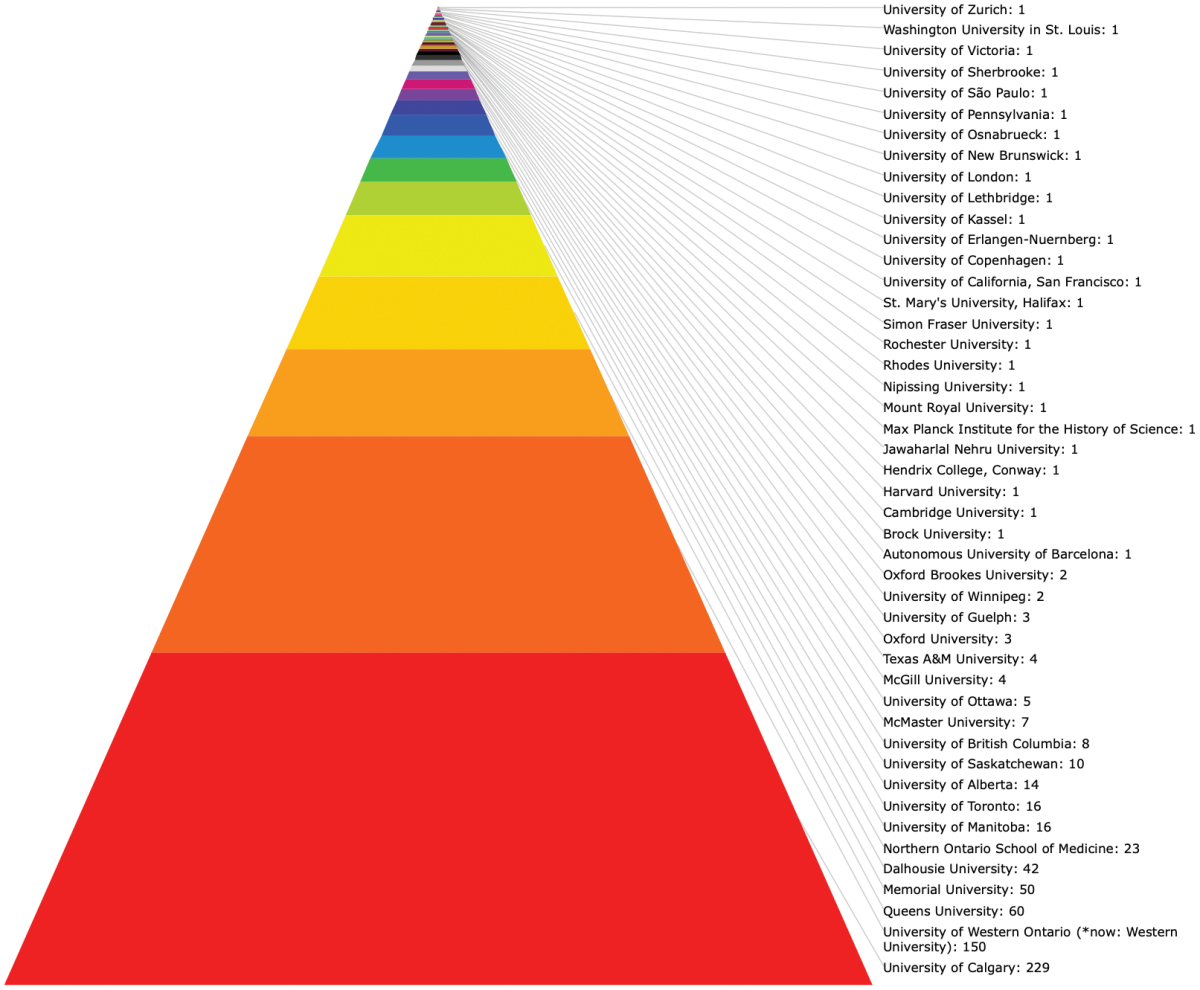
Percentage of Previous Prizes (Incl. Follower-up Status) Awarded to Students from Various Universities.

**Table 4.**
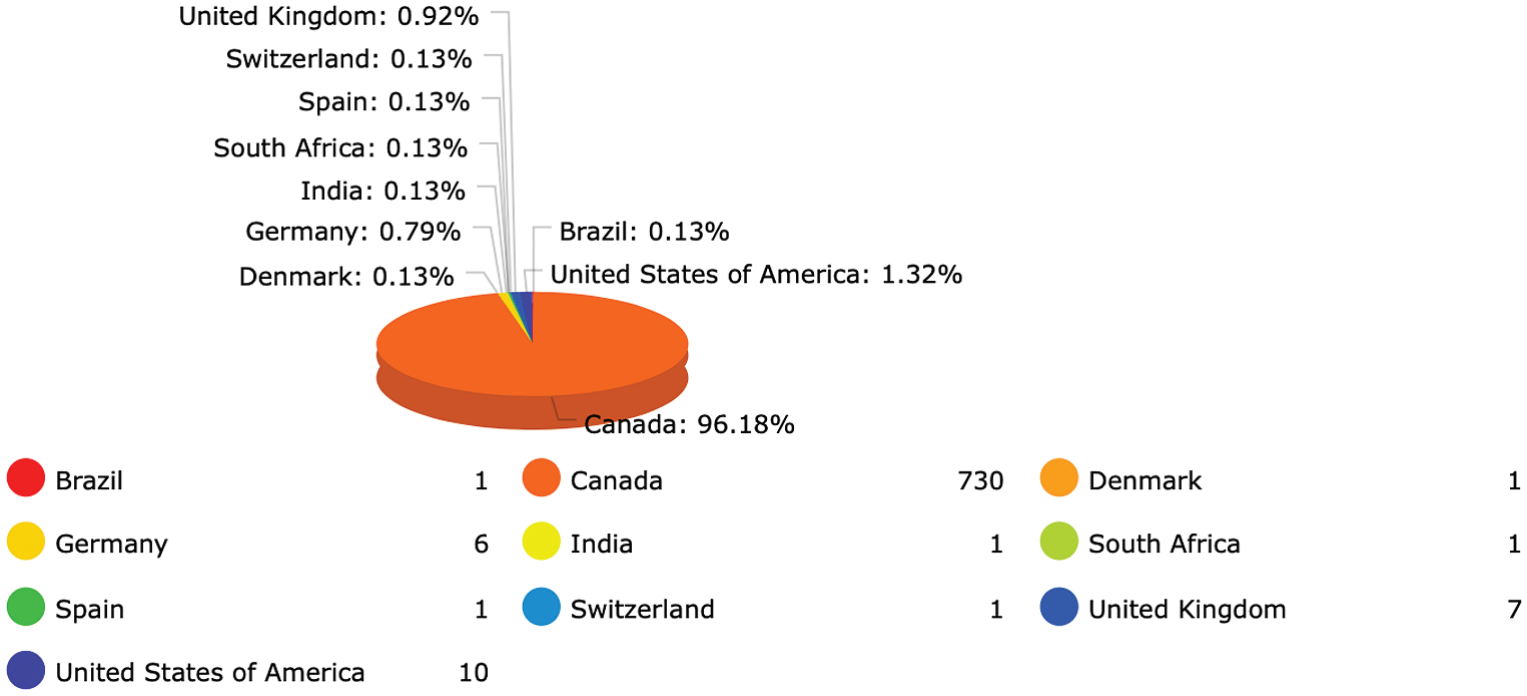
Percentage of Countries from which Speakers have Historically Come from to Attend HMDs.

The review process and the selection of high-quality papers is made by a local committee, which receives further input from the board of advisors, as well as from external historians of medicine. These selection processes have engendered a significant academic appeal for the HMDs at the UofC.^28^ The individual volumes of the Proceedings of the Calgary HMDs, appearing since 1996, provide a unique publishing format to students and clinical presenters from multiple perspectives, and include all conference keynotes.^29^

## INCORPORATING THE HISTORY OF MEDICINE INTO MEDICAL EDUCATION

Medical school instructors have long pondered the challenges of incorporating history of medicine teaching in student and clinical education, seeking ways to furbish insights into the humanities, social contextualization, and create ethical awareness in the curricula.^30^ In Germany, for example, history of medicine has had a continuous presence in the medical core curriculum since the 1920s.^31^ In North America, however, medical history has been integrated in a rather disjointed fashion into educational curricula, depending often on introductory lectures in discipline-based courses or elective courses for interested students.^32^ Of course, the administrative leadership of medical schools plays an important role in the determination where in students’ and clinicians’ schedules medical history content can be taught.^33^ At the relatively young medical school in Calgary, which hired its founding dean William Cochrane (1926–2017) in 1967 and was set up based on a tight three-year curriculum, it was decided that the history of medicine course had to take place as an elective course during the lunch hour and in evening workshops, while the goals of teaching centered around historical insights informing medical practice in the future as well as fostering professional virtues such as diagnostic humility, developing a broad knowledge basis, and awareness for the contingency of medical ethics issues and principles.^34^ The principles of the course and program had been founded on seeing medicine as a constituent part of science and technology studies, as well as gaining structural competency by examining the contingent and contextual development of medical practices and policies, including an understanding of the implications of political agendas for medical achievements and successes. Calgary, as a three-year school, differed from the standard four-year model – together with the Université de Sherbrooke (1961) and McMaster University (1969) at the time – with an emphasis on problem-based learning and early clinical immersion on the hospital wards from the very first day of classes.^35^ This early immersion of students in clinical training has translated into the historical revision of many clinical topics from day one of the course in medical history, including the physician’s role in modern hospitals, the influence of disease on medical thought and practice, as well as important changes in clinical diagnostics and nosology. These values of including historical perspectives in the training of young physicians are represented in the extended motto of the HOMHC program, which characterizes the UofC’s particular approach to the medical history:

(1) To attract students and faculty from interdisciplinary backgrounds and cross-departmental affiliation to engage in and contribute to the Program’s activities…; (2) to develop and expand the profile and standing of the Program within medical and interdisciplinary communities engaging in historical research and teaching medicine and health care across Canada and abroad; (3) to relate to the university, the local community and the public at large explaining and interpreting topics from the history of medicine, the life sciences and neuroscience…; (4) to conduct interdisciplinary teaching and research in the CSM and beyond by integrating views from the History and Philosophy of Science, Science and Technology Studies, Cultural and Media Studies.^36^

This directional statement emphasizes the importance of engaging with the social, cultural, political, and economic contexts of clinical training and medical knowledge. Today, the half-time validity of medical knowledge is getting increasingly shorter and 50% of medicine’s knowledge basis is revised every 5 years.^37^ Hence, it is important to broaden students’ and clinicians’ understanding of the time-dependence of medical diagnoses and pathologies, the contingency of medical knowledge, and the personal, cultural, and religious views which patients bring to the health care field. Ethical conundrums and legal questions in medicine are time-dependent and necessitate rational assessments that take the historical traditions honestly and soberly into account.^38^

Building a wider understanding of the central task and function of history in current medical curricula remains attractive to students, since it provides a valuable foundation to them.^39^ Its firm inclusion into medical education programs helps students appreciating the critical stance and ethical perspectives necessary to make sense of health care approaches in today’s fast-changing world.^40^ In Calgary, such an agenda has been sustained through the satisfactory endowment of a research chair first internationally advertised in 2007. Since the recruitment of an AMF/Hannah Professor in the History of Medicine and Health Care at the CSM, the teaching offerings in the history of medicine, psychiatry, and the life sciences have become more diversified.^41^ The History of Medicine and Health Care course has since received “a fresh hands on approach to integrating an understanding of the past with a vision of the future.”^42^ The help and support of the UofC’s Faculty of Arts, the Bachelor of Health Sciences Program, the Arts and Science Honours Academy, the Hotchkiss Brain Institute, and the O’Brien Institute for Public Health have been instrumental in curriculum design and in establishing meaningful research collaborations among faculties, programs, groups of clinicians, and public health researchers. At the UofC, the program development has resulted in a specific concept of medical history education, built upon the tradition in the city of Calgary of research and writing on the history of medicine as described above.^43^

Furthermore, a significant number of the history of medicine books from the former Calgary Associate Clinic were later donated to the UofC’s Health Sciences Library. There, the collection was merged with the “Dr. Peter J. Cruse Collection” and the “Mackie Family Collection in the History of Neuroscience.”^44^ Combined, they form the basis for a significant medical history research library.^45^ Students are attracted to history of medicine courses in part because of the active library sessions and field *practica* offered, which make extensive use of the Dr. Cruse Collection and Mackie Collection for their research projects and presentations concerning medical history.^46^

## HISTORY AND ACTIVITIES OF THE UOFC HISTORY OF MEDICINE AND HEALTHCARE PROGRAM

Enticing collaborations in research and education among physicians, medical scientists, historians, sociologists, and anthropologists has been, and continues to be, a foundational motivation for the UofC HOMHCP. To this end, meeting and exchange platforms, the history of medicine library collection, as well as interest groups and specialized colloquia, have been established to foster the interaction between the humanities and health sciences. Since 2012, the mutual interaction in the wider field of the medical humanities has also become institutionalized through the collaboration with other members of the CSM’s Health Care Humanities Committee.

In their previous article “Making the Case for History in Medical Education” in this journal, David S. Jones et al. have argued that they see both chances as well as challenges with integrating medical history within the medical humanities, while making “the case for history as an essential component of medical knowledge, reasoning, and practice.”^47^ While these authors emphasize the challenges posed by aligning the medical humanities with medical history, the Calgary experience has shown rather a positive interaction, particularly medical students’ participation, interdisciplinary contributions to mutual workshops, as well as research collaboration regarding knowledge-based and epistemology-oriented projects. The Health Care Humanities Committee coordinates educational opportunities for medical students, clinical residents, and health care professionals and furthers active participation in the use of health care humanities in education. One useful example is the self-directed electives opportunity during medical students’ clerkship periods that explore a problem in medicine while working closely with a health humanities preceptor, including the available historians of medicine, and using humanities tools and learning about patient-centered perception using narrative analysis.^48^ Such activities have led to an increasing collaborative network among professors in the Faculty of Arts with faculty in the CSM, Nursing, and Science, as international historians of medicine have previously advocated.^49^ Since my arrival in Calgary as the AMF/Hannah Professor in the History of Medicine and Health Care, I have worked with students in both the department of community health sciences and the history department, including much of graduate student training – both on the Master’s and the PhD level – as well as the supervision of scores of undergraduate students with honors theses, summer research students, and clinical residents. Medical students prepare for the annual HMDs to present their term projects to the conference audiences as part of their course requirements. The conferences attract over 300 students and faculty to attend or present abstracts on the History of Medicine and Health Care (see table 1).^50^ The UofC HOMHCP has two core components: History of Medicine courses and the annual HMDs. The courses run for two terms from fall to spring each year and most of the students present their historical research projects at the conferences. This provides students with excellent experiences in preparing their work for the field and ample networking opportunities with medical students from across the country, and often international students and faculty as well.^51^

The history of medicine courses are comprised of a series of lectures and seminars held on Mondays from 12:30 to 1:20 PM throughout the full academic year, which highlight particular themes, episodes, controversies, people, and events in the history of medicine. The types of history taught in these courses include the social history of medicine, the history of medical ideas, laboratory history, the relationship of the history of medicine with the history of science and the Science and Technology Studies (STS), elements from public health history, as well as the relationship between the history of medical technologies and modern media studies. These lectures and seminars, attended by medical students, residents, postdocs, and interested clinical faculty, provide an overview of history of medicine and are taught by both historians and medical faculty actively publishing in the history of pathology, pediatrics, geriatrics, public health, as well as complementary and alternative medicine.^52^ During the latter half of the course, there is abundant opportunity for students to give presentations on their topics and to engage in mutually profitable discussions. Students are provided with forums, which are also advertised to and attended by medical residents, clinical fellows, and postdocs, to develop their individual speaking skills at public events and share ideas about how they might arrange materials for their presentations. These interactions push medical students to emphasize the archival source bases of their projects, critically reflect on their practical experiences prior to or during their medical studies, and engage with real conversations about their future medical career aims in light of what they have learned about historical traditions and developments that have markedly changed the clinical context of physicians’ work today (including such aspects as economic pressures, the introduction of information technologies, and the burden of chronic diseases). Particularly, the series of discussion-based Thursday evening workshops from 17:30 to 19:20 PM, attended by the same *clientèle* as the Monday lectures, offer practical instruction and experience on various topics, ranging from skills for public presentation, academic discussion, history research methodology, to history of medical techniques with some hands-on experiential learning. The course offers thereby a good chance for medical students to establish informal relationships with faculty, who later serve as faculty preceptors in students’ disciplines of interest for individual research projects and even as collaborators on mutual publications.^53^ This also extends to informal exchanges between mentors and mentees about the reality of various clinical and research contexts, informed by senior mentors’ personal experiences from having lived through decades of medical transformations and changes in the health care system.

At the end of each academic year, the associate dean for undergraduate medical education receives a letter about each student’s participation in the course and spin-off activities, including archival work, presentations, and publications, as these are always seen as valuable additions to a student’s *curriculum vitae* as well as their electronic Canadian Resident Matching Service portfolios.^54^ Interested history students are eligible to take the history of medicine course too, while a numerical cap guarantees a ratio of two-thirds medical and health sciences students to one-third history students. If more than the eligible number of students sign up for the course, a selection criterion measures certain prior competency largely based around the ability to bring inter-professional perspectives to the course.^55^ A few weeks after the beginning of the course, interviews are set up for individual students to choose an area of research interest and be matched with a preceptor. It is expected that all students in the course must prepare and give a presentation of about thirty minutes on a topic they have researched, and they need to complete a methodological group assignment on “Heroes, Rogues, and Charlatans in the History of Medicine.”^56^ At the end of the course, all students are asked to submit a research paper of 15–20 pages on their topic, which may be later published in the *Proceedings of the Annual History of Medicine Days*.^57^ The social aspect of the HMDs can thereby not be underestimated in building a continuing community of history of medicine-endorsing and -enjoying physicians, faculty, and trainees, who locally and regionally continue to interact about the relevance of medical history in medical education over many years.

This content of the UofC’s HOMHCP is hence fully aligned with most of the strategies for history at medical schools in the US and Canada as pointed out in “Making the Case for History in Medical Education.” The concerns articulated in their article regarding resource competition between the medical humanities and history of medicine would not necessarily lead to antagonism but can also be overcome through mutual forms of collaboration and the emphasis of interdisciplinary strengths. Furthermore, Jones et al.’s focus on reframing history of medicine as a field that is based in science studies and history of science, discussing questions of paradigms, biopolitics, discourses, and postmodernism, can also be widened to include valuable public health perspectives, areas of comparative and global history, along with important aspects of intersectionality and indigeneity regarding the history of medicine and health care.^58^ And since 2015 such developments have indeed been renewed and refurbished in wider medical humanities and medical education perspectives, for example in teaching and research programs such as that at the UofC’s HOMHCP. Yet the main tenants of Jones et al., which encompassed several claims relevant for clinical medicine, have likewise been incorporated into the educational and research activities in UofC’s HOMHCP: “The burden of disease changes over time,” “the contingency of disease concepts,” “medical therapeutics are dynamic,” “knowledge is produced through socioeconomic processes,” “health inequalities have persisted,” “medicine has influenced notions of race, ethnicity, gender, sexuality, and class,” “medical education takes place across social disparities,” “medical technologies exist as part of broader social systems,” “the roles of physicians,” “hospitals are the by-product of political struggles,” “health-seeking behaviours have changed,” “medicine is one of many societal responses to disease,” and “historical study has shown that individuals’ experiences have changed over time.”^59^

Students could also submit abstracts on medical topics to the Royal College of Family Physicians of Canada for their student prize competition.^60^ And a number of presenters at HMDs have been invited to present their work at the Margaret Hutton Lectures of the AMA.^61^ Some outstanding students and clinician participants have sought additional opportunities to publish their manuscripts in high-end international history of medicine journals.^62^ They further submitted them to the William Osler Medal competition of the American Association for the History of Medicine.^63^ In the past, several participants gave external interviews on their topics to CBC Radio.^64^ Advanced students could choose history of medicine as an option in their research elective during the summer of the second year of the medical education program.^65^ Occasionally, students from other universities are residents in the program and work on specific research projects over the summer, while priority is given to students who investigate similar subjects as the Calgary faculty.^66^ Workplaces are provided during the summer season in both the departments of community health sciences and history, while residents are required to give one presentation on their topic to the program associates, sometimes resulting in publishable work.^67^ The following pursuits comprise the core objective of the program for students: the development of an understanding of the general history of medicine, the selection of a specific area of the history of medicine for more detailed study, the cultivation of a critical perspective on medicine and the doctor-patient relationship, and the development of historical research skills.

Although the Calgary History of Medicine and Health Care course has changed in nature and scope since it was started by Peter Cruse, students remain committed to the experience of the program, which offers them insight from various faculty members into their specific areas of interest.^68^ The intriguing character of the field is likewise reflected in the research exchanges during the Calgary HMDs, which have now grown into the only singular national event for students and trainees – strongly supported by the eight Canadian Hannah Chairs in the History of Medicine (many associated with the referenced medical history programs in the next section of this article).^69^ Canadian Hannah Chairs have been sending their own students and trainees or provided lectures to the program – at which both undergraduate, early graduate students, and postgraduate students in medical history can meet and actively practice humanism in medicine with likeminded peers.^70^ This gives testament to Cruse’s hope that medical history would elevate itself when he referred to the *aperçu*: “art always outlasts science.”^71^ One may find this further reflected in the outstanding publications by medical students that have derived from the UofC courses and the HMDs, with many Canadian, American, and international physicians having started their careers in or through the program.^72^ These activities certainly align with discussions by professional medical historians in North America and with the values promoted by the Association of American Medical Colleges. The program is undergirded by the belief that “history acts as a unifying force connecting a variety of scientific and humanistic disciplines and, by providing a historical perspective, serves to promote the student as a professional.”^73^

## CONNECTING CANADA’S HISTORY OF MEDICINE PROGRAMS

Comparatively few North American medical schools include medical historians and history of medicine courses. At the UofC’s CSM, the muse of history is nevertheless seen as integrative to medical teaching, research, and collaboration, as this case study submitted to *Clio in the Clinic* emphasizes. The current analysis of the local history of medicine program can offer useful insights into the proliferation of the program along with the available resources and history of medicine collections. Education studies have frequently examined history of medicine programs to elicit how medicine is perceived, understood, and taught with a critical perspective to its historical development and socio-cultural context. This article has examined the historical steps that led to the foundation of the UofC’s HOMHCP and its context in the reform education initiatives at this younger medical school in Canada. The support of external funding agencies, the AMF and the Hannah Foundation (now the AMS), was instrumental in bringing a medical history perspective into the educational setting in Calgary. Rather being wedded to contingent local settings, the UofC has tried to reach out to other medical schools, nursing schools, and history of medicine programs, to stimulate active exchanges and place history of medicine in a wider communicative network.

Further networking opportunities were woven into the fabric of many of the history of medicine courses, including exchanges with colleagues from clinical education, and interdisciplinary collaboration with other historians, philosophers, anthropologists, and ethicists, as well as inter-professional panels.

This collaborative community is locally supported by the planning committees, the award committees, and a pool of volunteering judges and conference moderators. The program is presently supported by Melanie Stapleton, a gastroenterologist as first co-chair and Lesley Bolton, a classicist, as second co-chair; Stephen Pow, who arrived from the Central European University, Budapest, Hungary, as an AMS-funded postdoc; sessional history instructor Susan McMahon as a research associate; and Marcia Garcia as the course and conference coordinator; along with physician-historian Robert Lampard holding an adjunct position in the department of community health sciences. Previous postdocs have included Fedir Razumenko from Saskatoon, Saskatchewan; Aleksandra Loewenau from Oxford, UK; Will Pratt from Edmonton, Canada; and Matt Oram from Christchurch, New Zealand. The program is administratively located in the CSM and can be found on the third floor of the Cal Wenzel Precision Health Building, while the Science, Technology, Environment, and Medicine Studies laboratory is housed on the eighth floor of the Faculty of Arts, and the History of Medicine Room in the library being located on the first floor of the HSC. The following individuals or administrators serve on the advisory committee: the chair is the associate dean, research (CSM), Bill Whitelaw as former HOMHCP chair, the head of the department of community health sciences, the head of the department of history, the associate dean for undergraduate medical education (CSM), the director of the O’Brien Institute for Public Health, the director of the Hotchkiss Brain Institute, classicist Peter Toohey, the dean of the Faculty of Arts, a student representative, and the CEO of AMS. The current state of the history of medicine curriculum at the UofC has further been exported to other schools, most visibly at the UofA’s medical school in Edmonton, where the history of medicine interest program was restarted and revitalized by Jamil Kassam, a historian trained at the University of Chicago with (see table 5) a specialization in the history of Arabic-Islamic medicine. Content from the UofC’s HOMHCP has been woven into the fabric of the Edmonton curriculum, and the course syllabi developed at the UofC been used as a basis for the teaching of medical students there, while their history of medicine interest group supports the sessions of the Calgary History of Medicine Society (CHOMS), comprising physicians, clinical faculty, as well as medical students and residents in Calgary with its own research contributions in an active and successful way.^74^

**Table 5:** History of Medicine-Related Course Offerings Across the University of Calgary.

HTST493.38	History of Medicine and Health Care I	Medical Students Year 1-2	Full Course
HTST493.39	History of Medicine and Health Care II	Medical Students Year 1-2	Full Course
No Number Assigned Yet	Elective Research Project Course	Graduate Students with Previous Health Care-Related Degrees	An MA in Humanities in Health Care is Currently in Planning (in Collaboration with the Humanities in Health Care Committee)
HTST791.20	Medicine and its Intersections: Historical Origins and Foundations of Western Medicine and Health Care	Graduate Students (including the MA in History and Philosophy of Science)	Full Course
MDCH680	Foundations of Population and Public Health	Graduate Students (Health Sciences)	1 Introductory Lecture on the History of Public Health
GRST601.62	A Social and Cultural History of Mental Health and Psychiatry since the Classical Period	Graduate Students (Classics and Religious Studies and MA in History and Philosophy of Science)	Full Course
HTST541.2	History of Medicine and Psychiatry in the Western Context	History, Philosophy, and Health Sciences Students Year 3-4	Full Course
ASHA501	The Nature of Research	Arts and Science Honors Academy Year 4	Full Course
HTST476	*Wonderful Life:* A Cultural History of the Biomedical Sciences	History, Philosophy, and Health Sciences Students Year 3-4	Full Course
NEURO421	Neuroscience: History, Ethics, and Society	Neuroscience and Psychology Students Year 2-3	33% of Full Course
HTST372	*Ways of Knowing*: Science, Technology, and Medicine in Historical Perspectives	History, Philosophy, and Health Sciences Students Year 2-3	Full Course
GRST323	Ancient Medicine and the Mind	Classics and Religious Studies Students Year 3	Full Course
GRST321	Ancient Technology	Classics and Religious Studies Students Year 3	Full Course
GRST211	Technical Terms of Medicine and the Life Sciences	Master of Pathologists’ Assistants and Classics and Religious Studies Students Year 2	Full Course

Reviewing the structure and function of the HOMHCP reveals similarities, differences, and cooperation features with other history of medicine programs that developed around the same time across Canada. Many students and trainees from these centres have attended the HMDs conferences in Calgary:

### Dalhousie University

Its program in the medical humanities is located in the faculty of medicine and was led for four decades by physician-historian Jock Murray, who had been the former dean of the faculty. The program is primarily addressed to undergraduate medical students. Staff members include medical humanities scholar Wendy Stewart, historian of medicine Ronald Stewart, Marc Gilbert as postdoc, and Ana Sardinha as administrator.

### McGill University

The department of social studies of medicine was founded in 1966 by Donald G. Bates (1933–2001). It is situated in the medical faculty, but members are cross-appointed in the departments of their home discipline. A lecture on the history of medicine and seminar-style block courses are taught in the medical faculty, yet most of the undergraduate teaching occurs within the disciplinary departments. McGill’s is a research-intensive department, staffed by architectural historian Annmarie Adams as chair, sociologist of science Alberto Cambrosio, bioethicist Jennifer Fishman, medical ethicist Phoebe Friesen, bioethicist Jonathan Kimmelman, medical ethicist Nicholas King, historian of medicine Thomas Schlich, anthropologist Margaret Lock, medical historian Andrea Tone, medievalist Faith Wallis, historian of medicine George Weisz, and anthropologist Todd Meyers.

### University of Toronto

The Institute for the History and Philosophy of Science and Technology was created in 1967 as a graduate studies and research institute. As an autonomous unit, it remains outside the medical faculty, with close relations to other UofT faculties. Academic staff include philosopher of science Brian Baigrie, philosopher of science Hakob Barseghyan, philosopher of science Joseph Berkovitz, historian of medicine Lucia Dacome, religious studies scholar Yiftach Fehige, historian of mathematics Craig Fraser, historian of biology Nikolai Kremensov, philosopher of sociology Mark Solovey, medical anthropologist Wen-Ching Sung, historian of biology Marga Vicedo, philosopher of biology Denis Walsh, historian of technology Rebecca Woods, and historian of physics Chen-Pang Yeang.

Furthermore, the other seven Hannah Chairs continue to send students and participate themselves regularly in the HMDs, as organized by the UofC’s own Hannah Chair.^75^ These are Darrel Manitowabi at the Northern Ontario Medical School, Shelley McKellar at the Schulich School of Medicine, Jenna Healey at Queen’s University, Edward Shorter at the University of Toronto, Ellen Amster at McMaster University, Susan Lamb at the University of Ottawa, and George Weisz at McGill.

A particular aim of many history of medicine programs is the reinterpretation of Western culture, medicine, and science in critical, epistemological, constructivist, and other perspectives through teaching and research in medicine.^76^ When taking the development of the UofC HOMHCP as an example, it becomes clear that the personal leadership of individual academics, historical contingencies, and resource availability have shaped this program too:

[The Introduction of the teaching of history of medicine] is unlikely to occur in a “top-down” fashion…, it is much more likely to occur in a “ground-up” fashion, whereby energetic, committed, politically adroit historians will take advantage of local circumstances and opportunities at their own institutions, succeed in their work, and thereby facilitate the growth and spread of the effort.^77^

History of medicine programs actively further interdisciplinary collaboration between departments, faculties, and beyond – and, what is perhaps one of the greatest benefits of all, teaching faculty are available to meet with individual students to discuss their interests, provide feedback on history of medicine and career path questions, and offer research preceptorships for projects. Most medical schools, with their rather large budgets, could afford to finance medical history positions and would derive significant profit from them. The study of the history of medicine is central to the training of future doctors – from day one of their classes, while offering a space for insightful reflection of their work on the clinical wards and clerkship periods – through the medical humanities and within the (ever) ongoing reform activities in medical education.^78^

## Footnotes

1 Ingrid Kaestner, “Two Institutions and Two Eras: Reflections on the Field of Medical History – An Interview: Owsei Temkin questioned by Gert Brieger,” *NTM Journal of the History of Science, Technology and Medicine* 7 (1999), 2-12.

2 Maria Szlatky, “A Vision of History: The Wellcome Institute for the History of Medicine,” *Medical History* 31 (1987), 102-103; Owsei Temkin, “Introduction,” in *The Double Face of Janus and Other Essays in the History of Medicine*, ed. Owsei Temkin (Baltimore: Johns Hopkins University Press, 1977), 3-37.

3 Frank Huisman and John Harley Warner, “Medical Histories,” in *Locating Medical History: The Stories and their Meanings*, ed. Frank Huisman and John Harley Warner (Baltimore: Johns Hopkins University Press, 2004), 7.

4 Editorial, “Jason A. Hannah and the Hannah Chairs for the History of Medicine,” *Bulletin of the History of Medicine* 52 (1978), 125-127.

5 Peter Cruse, “Preface,” in *The Proceedings of the 7th Annual History of Medicine Days held on 26-27 March 1998 in the Libin Theatre*, ed. UofC Faculty of Medicine (Calgary: University of Calgary, 1998), vii.

6 Scott Assen and Robert Lampard, “Dr. Peter Cruse and the Reduction of Surgical Site Infections,” *Canadian Journal of Infection Control* 30 (2015), 143-150. Among recent significant research scholarship by clinical residents from Calgary see, for example, Nathaniel Gamble, Benjamin Holler, Stephen Thomson, Stephen Murata, Frank W. Stahnisch and Guel A. Russell, “Is the Writing on the Wall for Current Medical Oaths? A Brief Historical Review of Oath Taking at Medical Schools,” *Medical Science Educator* 9 (2019), 603-607; Aravind Ganesh and Frank W. Stahnisch, “‘The Gray Degeneration of the Brain and Spinal Cord’ – A Story of the Once Favored Diagnosis With Subsequent Vessel-Based Etiopathological Studies in Multiple Sclerosis,” *Journal of Nervous and Mental Disease* 207 (2019), 505-514.

7 Peter Cruse, “Preface to the Collection of Papers” (lectures, history of medicine, UofC, AB, 1984–1985) (copy in the Cruse family archives).

8 Following a thematic outline of his mentor, Dr. Scarlett: William Musselwhite, *Earle Scarlett: A Study in Scarlett* (Toronto: Dundurn Press and Hannah Institute for the History of Medicine, 1991), 94.

9 Lorne S. Williams, “University of Calgary Marks 25 Years of Medical Training,” *Canadian Medical Association Journal* 153 (1995), 984-985.

10 Robert A. Lampard, “Peter Joseph Erasmus Cruse, MB ChB, FRCS (C), FRCS (Edin.), FACS (1927–2006),” in *The Proceedings of the 20th Anniversary History of Medicine Days Conference 2011*, ed. Aleksandra Loewenau, Kelsey Lucyk, and Frank W. Stahnisch (Newcastle upon Tyne: Cambridge Scholars Publishing, 2015), 1-30.

11 Editorial, “Henri Pierre Cruse, and Surviving Spouse, Aletta,” *Government Gazette – South Africa* 162 (1950), 125.

12 Luis H. Toledo-Pereya, “Historical Note: Christiaan Barnard,” *Journal of Investigative Surgery* 23 (2010), 72-78; Peter Cruse, “Surgical Wound Infection and Quality Control – The Last 25 Years” (lecture, 25^th^ anniversary of the Infection Research Laboratories, Dudley Road 10, 1989 (copy in the Cruse family archives).

13 John S. Garnder, “Earle Parkhill Scarlett of Calgary: 1896–1982,” *Canadian Bulletin of Medical History* 1 (1984), 107-114.

14 Robert Lampard, David Hogan, Frank W. Stahnisch, and James R. Wright, Jr., *Creating the Future of Health: The History of the Cumming School of Medicine at the University of Calgary, 1967–2012* (Calgary: University of Calgary Press, 2021), 78-79.

15 Jack Peach, *A Shelter from the Winds of Illness: Foothills Hospital, 1966–1991: Celebrating a Quarter Century* (Calgary: Foothills Hospital, 1991), 57.

16 D. Robert Wilson, William B. Parsons, Carl F. Bethke, and Heber C. Jamieson, *Early Medicine in Alberta, College of Physicians and Surgeons of Alberta: 75th Anniversary* (Edmonton: Alberta Medical Foundation, 1993), 67-71.

17 This approach proves to be still usable now, while some of the required scholarly readings today include: Larence I. Conrad, Michael Neve and Vivian Nutton, eds., *The Western Medical Tradition 800 BC to 1800 AD* (Cambridge: Cambridge University Press, 1995); Deborah Brunton, ed., *Medicine Transformed. Health, Disease and Society in Europe 1800–1930* (Manchester: Manchester University Press, 2004); Frank W. Stahnisch. *Medicine, Life and Function: Experimental Strategies and Medical Modernity at the Intersection of Pathology and Physiology* (Bochum: Projektverlag, 2012).

18 Calgary Associate Clinic, ed., *Historical Bulletin: Notes and Abstracts Dealing with Medical History* (Calgary: Calgary Associate Clinic, 1944–1957); Hugh Ernest MacDermot, “The Calgary Associate Clinic Historical Bulletin,” *Canadian Medical Association Journal* 78 (1958), 792.

19 See an overview provided by: Peter L. Twohig, “Recent Writing on Health Care History in Canada,” *Scientia Canadensis* 26 (2002), 7-28.

20 Jacques Robichon, “Meeting Highlights – Points saillants du congrès annuel,” *Annals of the Royal College of Physicians and Surgeons of Canada* 13 (1980), 8.

21 John B. Neilson and G. R. Paterson, *Associated Medical Services, Incorporated: A History* (Toronto: Associated Medical Services and the Hannah Institute for the History of Medicine, 1987), 211-216.

22 On further background of the historical funding initiatives of AMS, see in: Charles G. Roland, “The Magnate of Medical History: The Prickly Dr Jason A Hannah Established One of the First Health Insurance Schemes in Ontario, but He is Best Remembered for the Institute of the History of Medicine Founded in His Name [Hannah Institute for the History of Medicine],” *Medical Post, Chicago* 34 (1998), 18.

23 William A. Whitelaw, ed., *The Proceedings of the Annual History of Medicine Days held in the Libin Theatre* (Calgary: University of Calgary, 1999–2007), 9 vols.

24 Ian A. Cameron, “History of Medicine Days, Calgary 2003,” *Canadian Bulletin of Medical History* 20 (2003), 452.

25 Jock Murray, “Proceedings of the 10th Annual History of Medicine Days, University of Calgary,” *Canadian Bulletin of Medical History* 21 (2004), 185. Of particular worth mentioning is that the HMDs have been accredited for 11 years for Continued Medical Education credit points under Section 1 of the Framework of continuing professional development Options of the Maintenance of Certification program of the RCPSC (https://www.royalcollege.ca/rcsite/cpd/maintenance-of-certification-program-e). This enticed further clinicians to join the HOMHC program and has led to excellent systematic and requisite program evaluations demonstrating student buy-in, regarding the broadening of clinicians’ historical horizons, better understanding of the contingencies of current-day medical policies, and specific insights into topics in physicianship (including bedside manners, whitecoat ceremonies, and topics in medical ethics), fully in line with the Canadian Medical Education Directives for Specialists’ Roles Framework, adopted in 1996 (https://www.royalcollege.ca/rcsite/canmeds/canmeds-framework-e).

26 Frank W. Stahnisch, “Preface and Acknowledgements,” in *Selected Papers on the History of Medicine and Healthcare (2014)*, ed. William J. Pratt, Lesley Bolton, and Frank W. Stahnisch (Newcastle upon Tyne: Cambridge Scholars Publishing, 2019), xvii-xxiv.

27 Peter Cruse, “University of Calgary Students Keen to Revisit Medical History,” *Canadian Medical Association Journal* 156 (1997), 628.

28 Frank W. Stahnisch (with co-editors), *The Proceedings of the Annual History of Medicine Days Conferences – A Series in the History of Medicine and Health Care* (Newcastle upon Tyne: Cambridge Scholars Publishing, 2009–ongoing).

29 The volumes further contain the collated abstracts of the respective conferences for documentation purposes and are well-illustrated with diagrams and images pertaining to the history of medicine. Ian A. Cameron, “History of Medicine Days, Calgary 2004,” *Canadian Bulletin of Medical History* 22 (2005), 418-419.

30 Huisman and Warner, *Locating Medical History*, 1-30.

31 Heiner Fangerau and Maria Carla Gadebusch-Bondio, “Spannungen in der juengeren Medizingeschichte: Legitimations­strategien und Zielkonflikte – ein Beitrag zur Diskussion,” *NTM Journal of the History of Science, Technology and Medicine* 23 (2015), 33-52.

32 Jonathan Fuller and Margaret M. Olszewski, “Medical History in Canadian Undergraduate Medical Education, 1939–2012,” *Canadian Bulletin of Medical History* 30 (2013), 199-209.

33 For the Calgary situation, see in: Henry Mandin, Peter Harasym, Chris Eagle, and Mo Watanabe, “Developing a Clinical Presentation Curriculum at the University of Calgary,” *Academic Medicine* 70 (1995), 186-193.

34 Frank W. Stahnisch, “The Faculty of Medicine and its Response to the Changing Health-Care Context in the Province of Alberta, 1966–2016,” in *Fifty Years of Health Care Research at the University of Calgary in Alberta*, ed. Diana Mansell, Frank W. Stahnisch, and Paula Larsson (Calgary: University of Calgary Press 2020), 13-45.

35 William A, Cochrane, “Philosophy and Program for Medical Education,” *Canadian Medical Association Journal* 98 (1968), 500-505.

36 Cruse drafted the earlier mission statement for the program as “Seek out that which is noble in our past and make it a living ideal in our lives” (Calgary: University of Calgary and Health Care Program, 1997), 1.

37 John P. A. Ioannides, “Why Most Published Research Findings are False,” *Public Library of Science Medicine* 10 (2005), 1418-1422.

38 Joel D. Howell, “Some Thoughts on History and ‘Healing Relationships,’” *American Journal of Bioethics* 6 (2006), 80-82.

39 Andrew Dickson, John H. Read, John W. Dawson, and William A. Cochrane, “Planning for Medical Education at University of Calgary,” *Canadian Medical Association Journal* 100 (1969), 665-666. The appreciation by medical students and young clinicians is further highlighted in the evaluation comments returned at the end of each course as well as the Universal Student Ratings of Instructions, including responses such as that “the presentations were very well organized and very detailed,” that “the lectures were very knowledgeable and helpful for appreciating clinical practice changes,” and “it is important to use research ethics boards to approve all research projects,” ibid. (copies in the HOMHCP archives).

40 Aaron Michelfelder, “Preparing Medical Students for an Ever-Changing World,” *Health Progress* 95 (2014), 44-47.

41 Lampard, Hogan, Stahnisch, and Wright, *Creating the Future of Health*, ix.

42 Introduction slides (September 2009–ongoing), from the UofC’s *HTST493.38 course* (Calgary: University of Calgary, 2009–ongoing).

43 Nicola A. Bobey and Robert Lampard, “The Ram’s Horn: The Commonplace Book of Dr. Earle Parkhill Scarlett,” *Annals of the Royal College of Physicians and Surgeons of Canada* 30 (1997), 292-295.

44 Robert M. Gordon, “A Book Collector’s Perspective,” in *A Short History of Neurology: The British Contribution (1660–1910)*, ed. Frank Clifford Rose (Oxford: Butterworth Heinemann, 1999): 237-74. Many books of the Mackie Family Collection have now been digitized with the help of gracious external donations received for this purpose, greatly benefitting the research endeavors of medical students and clinician researchers alike (http://contentdm.ucalgary.ca/digital/collection/hstns/search/searchterm/Mackie%20Family%20History%20of%20Neuroscience/field/subcol/mode/exact/conn/and/order/title/ad/asc).

45 Nicole LeFebvre, “The Mackie Family Collection in the History of Neuroscience at the University of Calgary: Presenting a Critical Cataloguing Project of this New Acquisition in the Health Sciences Library,” in *The Proceedings of the 20th Anniversary History of Medicine Days Conference 2011: The University of Calgary, Faculty of Medicine, Alberta, Canada*, ed. Aleksandra Loewenau, Kelsey Lucyk, and Frank W. Stahnisch (Newcastle upon Tyne: Cambridge Scholars Publishing, 2015), 287-304.

46 National Library of Medicine, ed., *Directory of History of Medicine Collections* (Bethesda: National Library of Medicine, 1980–ongoing), https://hmddirectory.nlm.nih.gov.

47 David S. Jones, Jeremy A. Greene, Jacalyn Duffin, and John Harley Warner, “Making the Case for History in Medical Education,” *Journal of the History of Medicine and Allied Sciences* 70 (2015), 623-652, 623.

48 Several opportunities for interactions between the HOMHCP and the Health Care Humanities Committee emerge from the elective description online at: https://ucalgary.ca/groups/health-humanities/activities/med-440-self-directed-elective.

49 Howard I. Kushner, “The Art of Medicine – Medical Historians and the History of Medicine,” *Lancet* 372 (2008), 710-711.

50 William A. Whitelaw, “History of Medicine Days, Calgary 2004,” *Canadian Bulletin of Medical History* 21 (2004), 418-419.

51 Geoffrey Hudson, “Students attend History of Medicine Conference,” *Northern Passages* (Spring 2008), 4.

52 John V. Pickstone, “Medical History as a Way of Life,” *Social History of Medicine* 18 (2005), 307-323.

53 Cf. Stéphane R. Duchesne, Christopher Naugler, and James R. Wright, Jr., “Benjamin Terry and His Rapid Razor Section Method for Intraoperative Diagnosis,” *Journal of Medical Biography* 26 (2018), 156-164.

54 Brendan W. Munzer, Max Griffith, Whitney A. Townsend, and Jesse Burk-Rafel, “Medical Student- and Resident-Authored Publications in Academic Medicine from 2002 to 2016: A Growing Trend and Its Implications,” *Academic Medicine* 94 (2019), 404-411.

55 Paul N. Uhlig, Joy Doll, and Kristy Brandon, “Interprofessional Practice and Education in Clinical Learning Environments: Frontlines Perspective,” *Academic Medicine* 93 (2018), 1441-1444.

56 Jacalyn Duffin, *History of Medicine: A Scandalously Short Introduction*, 3rd. ed. (Toronto: University of Toronto Press, 2021), 1-6.

57 Pratt, Bolton and Stahnisch, ed., *Selected Papers from the 23rd History of Medicine Days Conference* (2014).

58 Jane Nicholas, “Body, Medicine, and Gender in Canadian History,” *Acadiensis* 44 (2015), 161-170.

59 Jones et al., “Making the Case for History in Medical Education,” 638.

60 For example, Malika Ladha and Aravind Ganesh received the related Charles Peter W. Warren History of Medicine Essay Prize. Editorial, “Dr. Charles Peter William Warren, M.A., M.B., B.Chir. (Cantab.), F.R.C.P, F.R.C.P.C.,” *Winnipeg Free Press* (14 May 2011), 1.

61 Award recipients of these AMA student lectureships included, for example, Steven Thomson, Scott Assen, Siddarth Shinde, Aravind Ganesh, and Sarah Erem, among many others.

62 Cf. Fedir Razumenko, “The Beginnings of the Canadian Cooperative Clinical Cancer Trials Program and the American Influences, 1962–76,” *Canadian Bulletin of Medical History* 37 (2020), 23-49; Anzo Nguyen and Frank W. Stahnisch, “From Interned Refugee to Neuropathologist and Psychiatrist,” *Journal of the History of the Neurosciences* 28 (2019), 351-360; James R. Wright, Jr., “The Radicalization of Breast Cancer Surgery: Joseph Colt Bloodgood’s Role in William Stewart Halsted’s Legacy,” *Bulletin of the History of Medicine* 92 (2018), 141-171.

63 Daniel Huang presented his topic at the 2019 HMDs conference in Calgary, receiving the Dr. Bill Whitelaw Award, and consecutively was awarded the Osler Medal in 2020 for his paper, entitled “Cyber Solace: Historicizing an Online Forum for Depression 1990–1999.”

64 Morgan Haigler, “Canada’s Forgotten Medical Past,” *The Gauntlet* 33 (19 March 2009), 1.

65 See the elective offers posted online at: https://ucalgary.ca/groups/health-humanities/activities/med-440-self-directed-elective.

66 In accordance with some principles shared in: Francine Smith, Peter Harasym, Henry Mandin; and Fritz Lorscheider, “Development and Evaluation of a Research Project Program for Medical Students at the University of Calgary, Faculty of Medicine,” *Academic Medicine* 76 (2001), 189-194.

67 Aravind Ganesh, “An Enduring Mystery – A Brief History of Multiple Sclerosis in Canada, 1850–1950,” *Alberta Doctor’s Digest* 37 (2012), 14.

68 David B. Hogan, “History of Geriatrics in Canada,” *Canadian Bulletin of Medical History* 23 (2007), 131-150.

69
https://www.ams-inc.on.ca/team/hannah-chair/.

70 George E. Thibault, “Humanism in Medicine: What Does It Mean and Why Is It More Important Than Ever?” *Academic Medicine* 94 (2019), 1074-1077.

71 Philipp M. Teigen, “Why Historians of Medicine are Wary of History of Bibliography,” *Canadian Bulletin of Medical History* 13 (1996), 181-186.

72 Previous program versions can be found online through the archives of the UofC’s HOMHCP at: https://www.ucalgary.ca/programs/history-medicine/hmd/past-hmds.

73 Erich H. Loewy, “Teaching the History of Medicine to Medical Students,” *Journal of Medical Education* 60 (1985), 692-695, 692.

74 A sense of the program activities in the History of Medicine at the University of Alberta in Edmonton can be gleaned from: https://www.ualberta.ca/medicine/programs/md/our-program/curriculum/electives-catalogue-year-1-2/history-of-medicine-across-worlds-and-time.html.

75 The past keynote speakers since 2008, for example, included Dawna Gilchrist (University of Alberta), Garland Allen (Washington University in St. Louis), Geertje Boschma (University of British Columbia), George Weisz (McGill University), Theodore M. Brown (Rochester University), Guel A. Russell (Texas Agricultural and Mechanical University), Thomas Schlich (McGill University), Peter L. Twohig (St. Mary’s University), Fernando Vidal (Autonomous University of Barcelona), Paul J. Weindling (Oxford Brookes University), Thomas Soederqvist (University of Copenhagen), Paul Potter (Schulich School of Medicine), Geoffrey Hudson (Northern School of Medicine), and Larry Zeidman (Loyola University Chicago Stritch School of Medicine).

76 Dorothy Porter and Frank W. Stahnisch, “Introduction,” in *Boundary Work and Trading Zones in the History of Medicine and Medical Humanities*, ed. Frank W. Stahnisch and Dorothy Porter (Salt Lake City: University of Utah Press, 2015), 4-24.

77 Kenneth M. Ludmerer, “The History of Medicine in Medical Education,” *Journal of the History of Medicine and Allied Sciences* 70 (2015), 656-660, 660.

78 Jon Arrizabalaga, “Does History Matter? Commentary on ‘Making the Case for History in Medical Education’,” *Journal of the History of Medicine and Allied Sciences* 70 (2015), 653-655.

