# Correction to: Making Medical History Relevant to Medical Students: The First Fifty Years of the Calgary History of Medicine Program and History of Medicine Days Conferences

**DOI:** 10.1093/jhmas/jrad010

**Published:** 2023-03-02

This is a correction to: Frank W Stahnisch, Making Medical History Relevant to Medical Students: The First Fifty Years of the Calgary History of Medicine Program and History of Medicine Days Conferences, *Journal of the History of Medicine and Allied Sciences*, 2023; jrac044, https://doi.org/10.1093/jhmas/jrac044

In the originally published version of this manuscript, there were several errors related to tables and captions. Table 1 should show the statistic chart from Table 3. The caption of Table 3 should read: “Percentage of Previous Prizes (Incl. Follower-up Status) Awarded to Students from Various Universities” and Table 3 should show the statistic chart from Table 4. The caption under Table 4 should read “Percentage of Countries from which Speakers have Historically Come from to Attend HMDs” and Table 4 should show the statistic chart from Table 1.

Publisher sincerely apologises for these errors that have now been corrected.

